# Sporadic fatal insomnia in a young woman: A diagnostic challenge: Case Report

**DOI:** 10.1186/1471-2377-11-136

**Published:** 2011-10-31

**Authors:** Karen M Moody, Lawrence B Schonberger, Ryan A Maddox, Wen-Quan Zou, Laura Cracco, Ignazio Cali

**Affiliations:** 1 Texas Department of State Health Services, 1100 West 49th Street, Austin, Texas 78756-3199, USA; 2 Centers for Disease Control and Prevention, Atlanta, Georgia, USA; 3 National Prion Disease Surveillance Center, Case Western Reserve University, Cleveland, Ohio 44106-7288, USA

## Abstract

**Background:**

Sporadic fatal insomnia (sFI) and fatal familial insomnia (FFI) are rare human prion diseases.

**Case Presentation:**

We report a case of a 33-year-old female who died of a prion disease for whom the diagnosis of sFI or FFI was not considered clinically. Following death of this patient, an interview with a close family member indicated the patient's illness included a major change in her sleep pattern, corroborating the reported autopsy diagnosis of sFI. Genetic tests identified no prion protein (PrP) gene mutation, but neuropathological examination and molecular study showed protease-resistant PrP (PrP^res^) in several brain regions and severe atrophy of the anterior-ventral and medial-dorsal thalamic nuclei similar to that described in FFI.

**Conclusions:**

In patients with suspected prion disease, a characteristic change in sleep pattern can be an important clinical clue for identifying sFI or FFI; polysomnography (PSG), genetic analysis, and nuclear imaging may aid in diagnosis.

## Background

Human prion diseases are rare, transmissible, invariably fatal neurodegenerative diseases that are characterized by the accumulation of a misfolded host protein, the prion protein, in brain tissue. They are classified into three main groups: sporadic, acquired, and genetic. Sporadic cases, with no known environmental source of infection, include sporadic Creutzfeldt - Jakob disease (CJD), the most common human prion disease, and sporadic fatal insomnia (sFI), one of the least common [1]. Acquired cases include iatrogenic CJD, acquired by medical or surgical procedures, and variant CJD, usually acquired from consuming beef products contaminated with the agent of bovine spongiform encephalopathy [1]. Genetic or familial cases are linked to a mutation on the prion protein gene, and include several subtypes of Gerstmann Sträussler Scheinker syndrome, familial CJD, and fatal familial insomnia (FFI) [2]. sFI shares a very similar phenotype to FFI, but is not associated with a mutation in the prion protein gene [3]. FFI is linked to the presence of a D (aspartic acid) to N (asparagine) variation at codon 178 (D178N) coupled with the methionine at codon 129 (129M) on the mutant allele of the prion protein gene (*PRNP*) [4]. The presence of valine at codon 129 (129V) coupled with the same D178N mutation is associated with a very different phenotype reminiscent of CJD [4]. sFI lacks the D178N *PRNP *mutation but appears to be invariably associated with methionine homozygosity at codon 129 of the *PRNP*, suggesting that 129M, either coupled with the D178N mutation or present in both alleles in the absence of the mutation is a requirement for the phenotypic expression of fatal insomnia [4].

Although Kawasaki and colleagues described a probable case of sFI in 1997, the disease was definitively established in 1999 by both Mastrianni et al and Parchi et al utilizing the term sporadic fatal insomnia [3,5,6]. Parchi and colleagues reported five such cases in subjects between the ages of 36 and 70 years (mean 50) with duration of illness ranging from 15 to 24 months (mean 17.8) [3]. An additional ten patients have been reported in the literature as sFI, expanding the age range to 30-74 and the disease duration to 13-73 months [6-13].

Like FFI, sFI is characterized pathologically by thalamic atrophy and clinically by disrupted sleep, autonomic dysfunction, and motor abnormalities including myoclonus, ataxia, dysarthria, dysphagia, and pyramidal signs [3]. Other clinical features consist of peculiar behaviors that can be mistaken for psychotic signs. Because the patients are deprived of sleep they may display drowsiness during the day which may be described as hypersomnolence unless the abnormal nocturnal sleep pattern is recognized by electroencephalogram (EEG) and/or polysomnography (PSG). The rarity of the disease can make the diagnosis of sFI challenging. To make clinicians more aware of an unusual presentation of prion disease and to demonstrate the importance of pursuing a thorough sleep history when prion disease is being considered, we describe the clinical and pathological details of a patient whose sFI diagnosis had not been considered antemortem.

## Case presentation

### Clinical findings

In February 2007, the Centers for Disease Control and Prevention (CDC) and the National Prion Disease Pathology Surveillance Center (NPDPSC) notified the Texas Department of State Health Services (DSHS) of a 32-year-old woman with an 18-month history of progressive neurological symptoms suggestive of CJD. (Table 1) Based on the medical record and her neurologist, her illness began in August 2005 with attention deficits and progressive memory loss. In June 2006, she demonstrated anisocoria and bizarre behavior, including talking incoherently to herself, and she was then referred to psychiatry. On a mini-mental state examination, she scored abnormally low in the measure of attention and calculation and she had reduced ability to repeat the names of three unrelated objects [14]. Later in 2006 she was described as being in constant motion, having unfocused hand gestures, and continued difficulty with ambulation. She was reported as alert, but confused, sad, and having difficulty with her thought process. Physicians caring for the case patient discussed the possibility of several diagnoses such as viral encephalopathy, paranoid schizophrenia, and subacute sclerosing panencephalitis, yet the overall etiology remained unclear. By February 2007, the patient was unable to ambulate and became bed-bound. She continued to demonstrate bizarre behavior, inability to follow commands, and unintelligible speech. The patient expired in June 2007, 22 months after the onset of illness.

**Table 1:** Progression of clinical signs and symptoms

Date	Clinical signs and symptoms
August 2005	Onset: age 31† Increased attention deficit† Progressive memory loss† Sleep disturbance‡

February 2006	Bizarre behavior‡ Sitting in chair making loud incoherent noises‡

April 2006	MRI - negative for intracranial abnormalities†

June 2006	Anisocoria† Increased agitation‡ Incoherent speech‡ Balance and gait difficulties‡ Talking to self† Referred to psychiatry† Decreased attention, registration and calculation†

July 2006	Electroencephalogram (EEG) - bilateral periodic epileptiform discharges†

August 2006	Flat affect† Continued decrease in attention, registration and calculation†

October 2006	Confused† Constant movement†

November 2006	Sleep enhancing medication prescribed‡ Unfocused hand gestures† Continued difficulty with gait†

January 2007	Akathisia -inner restlessness† Places arms and legs in sustained postures† Bizarre behavior†

February 2007	Bed-bound† Unable to ambulate† MRI - supratentorial parenchymal atrophy with no other acute intracranial findings†

March 2007	Cerebrospinal Fluid (CSF) 14-3-3 testing performed - result is not elevated† Awake most of the time‡

June 2007	Death: Age 33 Duration of illness: 22 months Autopsied tissue sent to National Prion Disease Pathology Surveillance Center

August 2007	Western blot revealed presence of abnormal protease resistant prion protein Immunohistochemical analysis revealed granular deposits as seen in prion disease MM2 sCJD, thalamic type consistent with "sFI"

† Information obtained from medical record

‡ Information obtained from family member

**Disclaimer: **The opinions expressed by authors contributing to this journal do not necessarily reflect the opinions of the Centers for Disease Control and Prevention or the institutions with which the authors are affiliated.

Over the course of her illness, she had EEGs, magnetic resonance imaging (MRI) studies, and cerebrospinal fluid (CSF) tests. The EEG study performed in July 2006 showed generalized slowing with bilateral periodic lateralized epileptiform discharges. A second EEG performed two to three weeks later was unsuccessful due to excessive movements of the patient. In April 2006, an MRI study was negative for intracranial abnormalities. Another MRI study was completed in February 2007 and it showed supratentorial parenchymal atrophy with no other acute intracranial findings. CSF studies performed in March 2007 were normal, including the amount of the 14-3-3 protein determined.

Because of the age of the patient and the potential for variant or iatrogenic CJD, in July 2007 an investigator from the DSHS (KMM) interviewed a family member to obtain additional information about the patient's travel history, past medical history, and the symptoms of the present illness. The patient had a history of travel outside the continental United States to Puerto Rico during 1995-96 where she had lived approximately one year. Her surgical history included two back surgeries for internal disc disruption and degenerative disc disease. An anterior lumbar discectomy with interbody fusion at L4-5 was performed in November 2000 utilizing cadaver donated bone and in August 2001 another fusion was performed at L5-S1 utilizing autologous bone. The donor of cadaver bone was pre-screened minimizing the possibility of iatrogenic transmission. There was no familial history of progressive neurological disease or dementia-like illness. The family member also confirmed the clinical history including the onset in August 2005 of progressive memory loss and, in February 2006, bizarre behavior that included the patient's sitting in a chair for hours making noises that progressively got louder.

Following preliminary autopsy results, the NPDPSC requested the DSHS re-interview the family to ask specifically about the patient's pattern of sleep. When questioned about insomnia, the family member recalled that the patient had experienced disturbed sleep at the time of her disease onset. The family member also reported that the patient's sleep pattern progressively deteriorated throughout her illness. Some nights, for example, the patient did not sleep. On other nights when she did appear to be sleeping, her sleep was intermittent. During nights that the patient did not sleep, she would roam the house at all hours, unable to calm down. By August of 2006, four hours was the maximum amount of sleep the patient would get in one stretch and at times she would go two to three days without sleep. Medications were prescribed to help her sleep but they were not beneficial.

### Genetic analysis

Sequencing of the PrP gene open reading frame revealed methionine homozygosity at codon 129, with no pathogenic mutation.

### Histological examination

**Figure 1:**
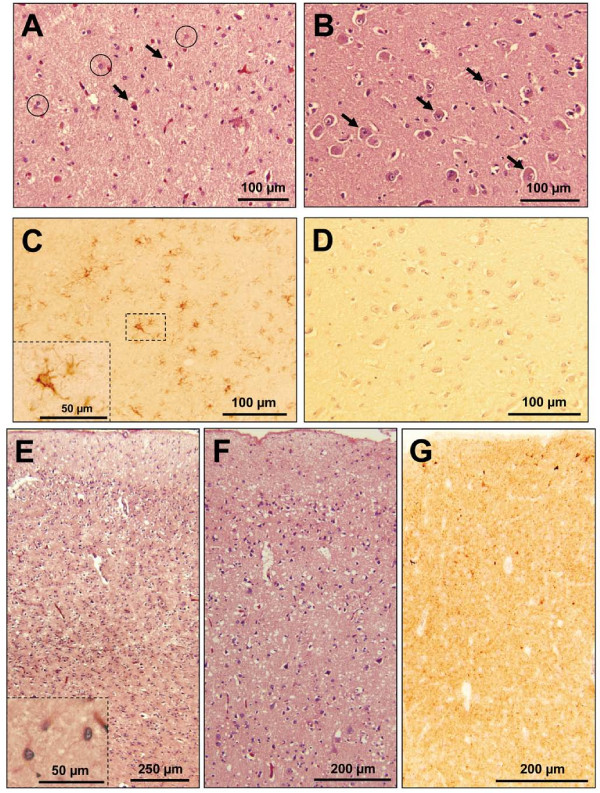
**Histology and immunohistochemistry**. **A**: Severe neuronal loss and astrogliosis of the mediodorsal thalamic nucleus in the present case. Neurons are indicated by arrows, reactive astrocytes by circles. **B**: For comparison, the same thalamic nucleus is shown in an age-matched subject without prion disease; neurons are indicated by arrows. **C**: Immunohistochemistry for glial fibrillary acidic protein (GFAP) reveals reactive astrocytic gliosis in the mediodorsal thalamic nucleus of the present case but not in a control subject of the same age without prion disease (**D**). **E**: Prominent astrogliosis in the frontal cortex. The inset (lower left corner) depicts three reactive astrocytes at higher magnification. **F**: Fine spongiform degeneration of the parietal cortex. **G**: Intense punctate or "synaptic" PrP immunostaining and sparse clusters of small granules in the cerebral cortex (parahippocampal gyrus; 3F4 antibody).

**Figure 2:**
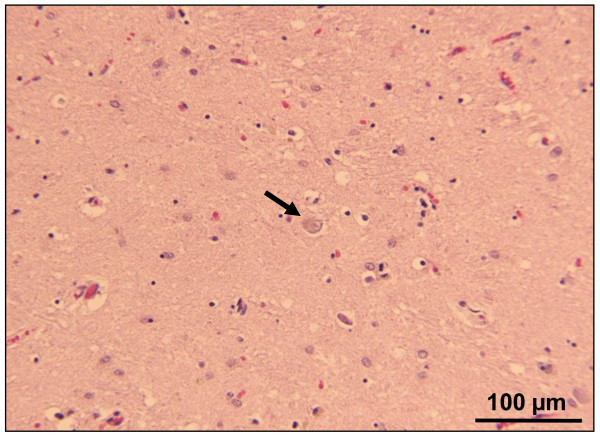
**Hematoxylin-eosin staining of the mediodorsal thalamic nucleus**. As seen in the present case, the hematoxylin-eosin staining of the mediodorsal thalamic nucleus also shows severe neuronal loss in a fatal familial insomnia (FFI) control case. The arrow indicates a thalamic neuron.

The mediodorsal and pulvinar thalamic nuclei along with the inferior olives showed severe neuronal loss and astrogliosis but no spongiform degeneration (SD) (Figure 1A-D and Figure 2). Astrogliosis with possible neuronal loss and superficial non-specific spongiosis affected particularly the frontal cortices while typical fine SD was present in other cortical regions, including parietal and temporal cortex (Figure 1E and 1F). With the exception of the presence of some SD in the molecular layer of the hippocampal formation, the hippocampus, basal ganglia, and cerebellum were much less affected than the cerebral cortex. Torpedoes, fusiform swellings of the Purkinje cell axons, were detectable in the granule cell layer of the cerebellum. Except in the hippocampus, where only the molecular layer was even weakly stained, immunohistochemical evaluation for the prion protein (PrP) in the cerebral cortex demonstrated intense staining in a predominantly 'synaptic' pattern with occasional small clusters of coarse granules (Figure 1G). Meanwhile, the basal ganglia, thalamus, and cerebellum were just faintly stained.

### Molecular study

**Figure 3:**
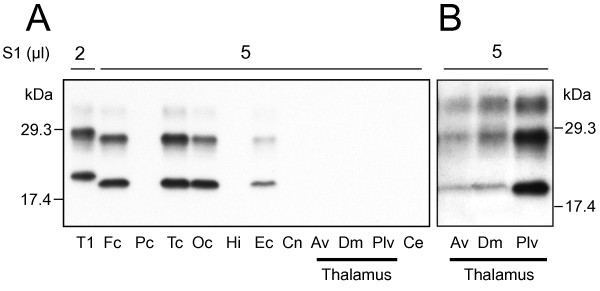
**Western blot analysis**. **A**: The unglycosylated fraction of PrP^res ^shows a gel mobility of approximately 19 kDa matching PrP^res ^type 2 in each brain region examined. S1 (μl): volume of brain supernatant (see Methods) loaded into the gel; T1: PrP^res ^type 1 (20 kDa) from a case of sCJD with genotype 129MM used as control. **B**: Western blot showing PrP^res ^from the thalamic nuclei indicated after probing with the 1:4,000 concentration of 3F4 compared to 1:40,000 in A. Fc: frontal cortex; Pc: parietal cortex; Tc: temporal cortex; Oc: occipital cortex; Hi: hippocampus; Ec: entorhinal cortex; Cn: caudate nucleus; Av: anterior ventral thalamic nucleus; Dm: mediodorsal thalamic nucleus; Plv: thalamic pulvinar; Ce: cerebellum.

High resolution and standard Western blot analyses of the abnormal and Protease K (PK)-resistant PrP (PrP^res^) from 19 brain regions invariably disclosed the presence of PrP^res ^type 2, which, however, varied in amount according to the location (Figure 3A). Of the two main types of unglycosylated misfolded prion protein fractions used as biochemical surrogate markers in prion diseases, PrP^res ^type 2 protein is slightly lighter than the PrP^res ^type 1 as demonstrated by their electrophoretic mobility on Western blots. The highest concentration of PrP^res ^was observed in most cerebral cortical regions such as frontal, temporal (including entorhinal), and occipital cortices, but it was minimal in the hippocampus. Variations in amount were also detected within the same cortical region (i.e. superior, middle, and inferior frontal gyri: data not shown). Minimal amounts of PrP^res ^were demonstrated in the caudate nucleus and in the three thalamic nuclei examined: anterior ventral, mediodorsal, and pulvinar (Figure 3A). No PrP^res ^was detected in the cerebellum (Figure 3A). Detectability of PrP^res ^in the thalamic nuclei was enhanced either by increasing the concentration of the antibody 3F4 ten-fold or by precipitating PrP^res ^with sodium phosphotungstate. (Figure 3B and data not shown). The ratios of the three PrP^res ^glycoforms, (diglycosylated, monoglycosylated, and unglycosylated) in the cerebral cortex and in the pulvinar, the only thalamic nucleus where PrP^res ^could be assessed accurately, were 15:42:43 and 28:38:34 respectively.

## Discussion and conclusions

The clinical diagnosis of prion disease in patients with signs of neurodegenerative diseases can be aided by paying particular attention to aspects of the patient history, including the patient's travels, past surgery, neurodegenerative illnesses in the family, and to possible changes in the patient's sleep pattern. Ancillary tests for suspected prion disease often include an EEG, MRI, and the measurement of CSF 14-3-3 protein, but these tests are typically unrevealing in cases of sFI. They also include genetic testing to detect possible *PRNP *mutations and to determine the genotype at codon 129 of the *PRNP*. Either valine or methionine can normally occur at the codon 129 of the *PRNP *and this polymorphism can strongly influence many aspects of human prion disease, including the disease phenotype and the susceptibility of a host to a prion infection. If FI is a diagnostic consideration, potentially helpful additional tests include PSG and nuclear imaging to demonstrate reduced tracer uptake in the thalamus [15,16]. Finally, pathological examination of brain tissue at autopsy is the definitive way to confirm the presence and type of prion disease.

For the patient described in this report, her long duration of illness and young age at onset are unusual for the most common subtype of prion disease, sporadic CJD [17]. Other forms of CJD were considered but determined to be extremely unlikely. Although this young patient showed signs of psychiatric illness at the beginning of her disease consistent with variant CJD (vCJD), these signs did not precede her noticeable deficits in attention and memory and she had not traveled to any country where transmission of vCJD was known to occur.

Iatrogenic CJD has been associated with a number of medical procedures. However, it is not known to be linked with receipt of a bone transplant. Furthermore, the donor of the bone transplant received by our patient had been pre-screened providing greater assurance of the absence in the donor of an infectious or neurological illness [18]. This patient also had no family history of neurodegenerative illness.

The history of insomnia was not in the medical chart nor was a sleep study or nuclear imaging study performed [7]. The neuropathological studies of the brain tissue demonstrated atrophy of the patient's thalamus, the neuropathological signature of both FFI and sFI, which then prompted the interview with a family member about the patient's sleep patterns. The diagnosis of sFI was made at autopsy based on the pathological evidence and the results of the genetic testing indicating the absence of a *PRNP *mutation. The history of progressively worsening insomnia is characteristic of sFI and underscores the importance of taking a careful history of possible changes in the patient's sleep pattern when evaluating an illness suggestive of a prion disease, particularly if the illness exceeds 12 months in duration.

The consistency of the association of sleep-wake disturbances with FFI and sFI has been recently challenged. Zarranz *et al *have examined the sleep disorder in 23 symptomatic carriers of the D178N mutation both homozygous for methionine (D178N-129MM) and methionine/valine heterozygous (D178N-129MV) [19]. Eleven of these patients were reported not to have insomnia. However, only two of these patients had a PSG study that is essential to rule out the presence of insomnia often difficult to detect clinically especially in the D178N-129MV patients. In both these patients PSG examination did reveal a severe sleep disorder compatible with FFI. The authors also claim that the clinicopathological phenotype was that of CJD rather than FFI in eleven of these 23 patients. However, autopsy examination of the brain essential to exclude the thalamic atrophy characteristic of FFI was carried out in only four of these eleven subjects and the histology of the thalamus is not described.

Combined, the studies of Landolt *et al*, Taratuto *et al *and La Morgia *et al *raise the issue of a wider prevalence of sleep disorder in prion diseases that deserves further study [20-22]. Landolt *et al *reported the presence of sleep-wake symptoms in all of seven patients with proven sCJD. However, the histology of the thalamus was examined in only four of the seven subjects and in a semi-quantitative fashion which regrettably did not include the assessment of the neuronal loss and the study of the thalamus in cases of sFI and FFI as positive controls of degree of thalamic atrophy. Furthermore, impairment of the autonomic system, a prominent component of the FFI phenotype, was not investigated in these cases [20]. These considerations are relevant also to the report of Taratuto *et al *of the presence of sleep impairment similar to that of FFI in a subject with the E200K-129MM mutation [21]. However, in this case the thalamus was fairly severely involved with gliosis and neuronal loss. Finally, La Morgia *et al *observed a sleep disorder similar to that of FFI and sFI in a case of sCJDVV2 with severe thalamic involvement by H-MR spectroscopy and detectable neuronal loss at histological examination [22].

Compared to the previous reports of sFI, the present case shows at least four major similarities: i) the presence of type 2 PrP^res^; ii) greater amount of PrP^res ^in the cerebral cortex compared to that in the sub cortical regions [3,10]; iii) glycoform ratios in the cerebral cortex and pulvinar that differ from that reported in FFI; and iv) no detectable PrP^res ^in the cerebellum. These findings along with the prominent thalamic atrophy and clinical evidence of sleep impairment definitely justify the classification of the present case as sFI.

Recently a case of alleged sFI has been reported showing the presence of PrP^res ^type 1 (rather than type 2 as in the present and other cases of sFI); the largest amount of PrP^res ^in the mediodorsal thalamic nucleus, and a glycoform ratio characterized by the relative prevalence of the diglycosylated PrP^res ^isoform similar to that of FFI [23]. If confirmed, this case indicates that, as in sCJD in general, occasional and unexplained phenotypic variations have to be expected in sFI. Finally, the severe neuronal loss of the anterior ventral and mediodorsal thalamic nuclei which contained relatively low amounts of PrP^res ^raises the issue of whether other isoforms of neurotoxic PrP such as protease-sensitive PrP are present in the thalamic nuclei in sFI.

## Materials and methods

### Reagents and antibodies

Proteinase K (PK), sodium phosphotungstic acid (NaPTA), N-Lauroylsarcosine sodium salt (sarkosyl), and phenylmethylsulfonyl fluoride (PMSF) were purchased from Sigma Chemical Co. (St. Louis, MO, USA). Benzonase nuclease was purchased from Novagen (Gibbstown, NJ, USA). Reagents for enhanced chemiluminescence (ECL plus) and the horseradish peroxidase-conjugated antibody were produced by Amersham Biosciences (Piscataway, NJ, USA). The 3F4 monoclonal antibody (mAb) was used against PrP residues 106-110 [24].

### Brain samples

Human brain tissues were obtained at autopsy and stored at -80°C. Samples were taken from 19 different brain regions: superior, middle, and inferior gyri of the frontal and temporal cortices, the middle gyrus of the parietal cortex, visual and non-visual occipital cortices, entorhinal and hippocampal cortices, basal ganglia (caudate nucleus, putamen, globus pallidus), substantia nigra, thalamus (anterior ventral, mediodorsal and pulvinar nuclei), and cerebellum (hemispheres).

### Molecular genetics

DNA was extracted from frozen brain tissues in all the cases, and genotypic analysis of *PRNP *coding region was performed as described [25].

### Histopathology and PrP immunohistochemistry

Histopathology and PrP immunohistochemistry were performed as described [26]. The sections were deparaffinized, rehydrated, and immersed in TBS-T. Endogenous peroxidase was blocked by Envision Flex Peroxidase Blocking Reagent (Dako) for ten minutes and washed. For 3F4 immunostaining only, sections were completely immersed in 1.5 mmol/L hydrochloric acid and microwaved for fifteen minutes. The slides were either incubated with GFAP 1:12000 (Sigma-Aldrich, St. Louis, MO) or 3F4 1:750 for one hour, washed, and incubated with Envision Flex/HRP polymer for 30 minutes (Dako). Envision Flex DAB (Dako) was used to visualize the immunoreactivity.

### Preparation of tissue homogenates and proteinase K digestion

Brain homogenates (10% w/v) were prepared in lysis buffer with 100 mM TRIS-HCl (100 mM NaCl, 10 mM EDTA, 0.5% NP-40, 0.5% sodium deoxycholate, 100 mM Tris-HCl, pH 8.0) and centrifuged at 1000 × *g *for five minutes to collect the supernatant (S1). Homogenates were incubated with 5 Unit/ml (U/ml) PK [48 Units/mg specific activity at 37°C, with 1 U/ml equal to 20.8 μg/ml PK] at 37°C for one hour, and then stopped by the addition of 2 mM PMSF. Samples were mixed in an equal volume of 2 × sample buffer (6% SDS, 5% β-mercaptoethanol, 20% glycerol, 4 mM EDTA, 125 mM Tris-HCl, pH 6.8) and boiled for ten minutes.

### Enrichment of PrP by sodium phosphotungstate (NaPTA) precipitation

Precipitation of PrP aggregates by NaPTA was conducted as described with minor modification [27]. Briefly, 10% (w/v) homogenates from brain were prepared in PBS lacking Ca^2+ ^and Mg^2+^. The samples were centrifuged at 1000 × *g *for 10 minutes at 4°C and 0.5 ml of supernatant was then mixed with an equal volume of 4% (w/v) sarkosyl prepared in PBS, pH 7.4, and incubated for 10 minutes at 37°C. Each sample was adjusted to final concentrations of 50 U/ml benzonase and 1 mmol/L MgCl_2 _and incubated for 30 minutes at 37°C. Aliquots were adjusted with 81.3 μl of a stock solution containing 4% (w/v) NaPTA and 170 mmol/L MgCl_2 _at a final concentration of 0.3% (w/v) NaPTA. Samples were incubated at 37°C for 30 minutes before centrifugation at 16,000 × *g *for 30 minutes. After isolation of the supernatant, the pellet was resuspended in 0.1% sarkosyl prepared in PBS, pH 7.4 for Western blotting.

### Western blotting

Proteins were separated by both non-commercial, home-made 15% Tris-HCl, 20 cm-long SDS-PAGE gels and 15% Tris-HCl Criterion precast gels (Bio-Rad, Hercules, CA). Proteins were then transferred to PVDF membrane (Immobilon-P; Millipore) for two hours at 60 V. The 3F4 antibody was incubated for two hours at room temperature (1:40,000 and 1:4,000). After incubation with horseradish peroxidase-conjugated sheep anti-mouse IgG at 1:3000, the PrP bands were visualized on Kodak film (Eastman- Kodak, Rochester, NY) by the ECL Plus (GE Healthcare, Fairfield, CT) as described by the manufacturer. Densitometric analysis was performed with UN-SCAN-IT gel 5.1.

## Consent

Written informed consent was obtained from the patient's next of kin for publication of this case report. A copy of the written consent is available for review by the Editor-In-Chief of this journal.

## Declaration of Competing interests

The authors declare that they have no competing interests.

## Authors' contributions

KMM conceived of the study, participated in the design, drafting, and revision of manuscript, conducted interviews and provided intellectual content. LBS made substantial contributions in the analysis and interpretation of data and was involved with drafting and revising it critically for important intellectual content. RAM contributed interpretation of data and critical revision of the manuscript. WQZ conceived of the study, contributed to analysis and interpretation of data, and critical revision of the manuscript for important intellectual content. LC contributed interpretation of data and critical revision of the manuscript. IC participated in acquisition of data, analysis and interpretation of data, drafting and revising the manuscript. All authors read and approved the final manuscript.

## Pre-publication history

The pre-publication history for this paper can be accessed here:

http://www.biomedcentral.com/1471-2377/11/136/prepub
